# Research on the impact of economic stress on young adults’ marital intentions from the perspective of social exchange theory: a study on the mediating role of psychological capital

**DOI:** 10.3389/fpsyg.2026.1758280

**Published:** 2026-03-19

**Authors:** Jihui Liu, Yuanyuan Shao, Yan Qiao, Fang Xu

**Affiliations:** College of Humanities and Social Sciences, Hebei Agricultural University, Baoding, China

**Keywords:** economic pressure, impact study, marital intention, psychological capital, social exchange theory

## Abstract

**Introduction:**

Drawing on social exchange theory, this study examined the impact of economic pressure on young people’s marital intentions, with a focus on the mediating role of psychological capital.

**Methods:**

This study used Mplus 8.0 structural equation modeling and SPSS 26.0 to analyze questionnaire data. Specifically, descriptive analysis was conducted on control variables such as sex, age, and education using SPSS, and differential analysis and reliability testing were performed on each variable; Mplus was used to compare the validity of various variables through model competition, and the relevant hypotheses of this study were also validated.

**Results:**

The results indicated a significant negative correlation between economic pressure and marital intentions (*β* = −0.411, *p* < 0.01), as well as between economic pressure and psychological capital (*β* = −0.392, *p* < 0.01). Conversely, psychological capital was positively correlated with marital intentions (*β* = 0.529, *p* < 0.01). Furthermore, psychological capital partially mediated the relationship between economic pressure and marital intentions.

**Conclusion:**

These findings suggest that economic pressure may diminish marital intentions in young adults partly by depleting psychological capital. The study highlights the importance of enhancing psychological resources in interventions aimed at supporting young people’s marriage decisions, offering practical implications for policymakers and mental health practitioners working with young adults.

## 1 Introduction

Marital intention refers to the subjective inclination of unmarried young people regarding whether they will marry in the future, representing their evaluation of the value of marriage and their personal feelings toward it (Li et al., 2022). However, as society continues to evolve, marriage behaviors among young people have exhibited new trends and characteristics. The rates of late marriage and non-marriage have risen rapidly. In 2023, there were 7.682 million marriage registrations nationwide; by 2024, this number had decreased by 1.576 million, a drop of 20.5%. In 2023, the average age of first marriage in China was 30.6 years for men and 28.7 years for women. In 2024, the average age of first marriage for men was 29.38 years and for women 27.95 years. In 2025, the average age at first marriage is projected to reach 27.5 years for women and 29.7 years for men by 2025. Before the pandemic (in 2020), the average age of first marriage in China was 28.7 years. Overall, the average age of first marriage in China after the pandemic was slightly higher than before the pandemic. This trend has gradually affected the well-being and development of individuals, families, and society, posing a serious challenge to the long-term balanced development of the population (Yang and Shi, 2024). It has also drawn considerable attention from both the public and academia. A review of previous literature reveals that the importance of resources for individuals and families has become increasingly prominent amid socioeconomic development, with economic factors being regarded as one of the core variables influencing young people’s marital intentions (Miao and Huang, 2022). However, existing research still focuses largely on the direct exchange of material resources, lacking sufficient analysis of the underlying psychological mechanisms.

Economic pressure refers to the psychological strain individuals or families experience when their available economic resources are insufficient to meet perceived financial needs and obligations. It is significantly correlated with an individual’s mental health. In China, the official poverty line is set at an annual income of 3,000 yuan, highlighting the objective threshold below which economic pressure becomes particularly acute. When economic pressure increases, it can trigger a series of negative emotional reactions, such as anxiety and tension, in individuals (Drentea and Reynolds, 2015). This anxiety, stemming from resource scarcity, amplifies the psychological burden and threshold perceptions of young people, leading them to assess the standards required for marriage more pessimistically. Consequently, their willingness to marry is diminished, discouraging them from entering into marital relationships (Jang and Cho, 2025). Based on the above analysis, this study adopts the perspective of social exchange theory to investigate the impact of economic pressure on young people’s marital intentions. American scholar Luthans et al. (2007) introduced the concept of “psychological capital, “defining it as a positive psychological state exhibited by individuals during their growth and development, which can buffer the impact of external stressors (Avey et al., 2009). This raises several questions: How does economic pressure affect an individual’s psychological capital? What implications does this have for young people’s marital intentions? And through what pathways does this mechanism operate? These questions will be explored in the following sections.

## 2 Theory and hypotheses

### 2.1 Relationship between economic pressure and marital intention

Current research indicates that unmarried young people perceive marriage as a “high-risk decision” that requires a careful assessment of benefits and costs (Li et al., 2025). From the perspective of social exchange theory, marriage represents a distinct form of social exchange. Traditional explanations have primarily focused on the exchange of material resources. Objectively, the increasing demand for such resources makes it difficult for many individuals to meet the material threshold for marriage, thereby diminishing their marital intentions. Subjectively, the prerequisite for making a marriage decision is the belief that one’s own resources are sufficient to meet marital demands, or that the anticipated gains justify the personal investment required. Economic resources, as a core component, can generate economic pressure that triggers negative emotions—such as anxiety, depression, and irritability—which in turn affect individuals’ judgment and assessment of exchange behaviors, ultimately hindering their decision to marry. Studies have shown that economic pressure can heighten individuals’ sensitivity to “good resource” traits in potential partners as a way to compensate for their own resource deficiencies. This strengthens the emphasis on partner conditions, raises the threshold for entering a marital relationship, and consequently leads individuals to systematically delay or forgo marriage altogether (Tian et al., 2019). Relevant surveys on the marital intentions of unmarried groups have also found that those from more affluent family backgrounds tend to exhibit stronger marital intentions (Li et al., 2022). Furthermore, prolonged economic pressure can negatively impact young people’s self-esteem and mental health. Research by Luerssen et al. (2017) indicates that individuals with low self-esteem often adopt “self-protection” strategies when entering intimate relationships (Elahi et al., 2018), viewing emotional expression as a form of “self-risking” behavior. This, in turn, diminishes their marital intentions and leads them to avoid entering a marital relationship.

Based on the foregoing analysis, this study posits the following hypothesis:

*H1*: Economic pressure is negatively associated with marital intentions.

### 2.2 The mediating role of psychological capital

Psychological capital refers to a positive psychological state exhibited by individuals during their growth and development. It is a psychological quality with both trait-like and state-like characteristics that helps individuals cope with adversity and stress (Conger and Donnellan, 2007). As Sendhil Mullainathan noted in Scarcity: Why Having Too Little Means So Much, concerns about one’s economic resources can occupy substantial cognitive resources, thereby weakening an individual’s psychological quality and depleting their psychological capital (Mullainathan and Shafir, 2013). Existing studies have shown that prolonged economic pressure can lead individuals to feel anxious about future prospects, trigger stress responses, increase psychological strain, and result in adverse behavioral changes (Lv et al., 2024). This vicious cycle continuously depletes an individual’s psychological capital, diminishing their self-efficacy and hope in dealing with future challenges on the one hand, while reducing their optimistic attitude toward life prospects and their resilience on the other.

Based on the above analysis, this study proposes the following hypothesis:

*H2*: Economic pressure has a significant negative impact on psychological capital.

As a key internal psychological resource, psychological capital influences individuals’ marital decisions through both cognitive and emotional pathways. From a social cognitive perspective, individuals with higher levels of psychological capital tend to assess marriage more positively, viewing it as a partnership for mutual growth rather than merely a burden. Such individuals possess greater self-confidence in their ability to navigate marriage and believe that through mutual effort, they can achieve shared life goals. Furthermore, psychological capital affects marital decisions through its emotion regulation function. Individuals with sufficient psychological capital are able to draw on resources such as hope and optimism to maintain positive expectations when facing uncertainty, thereby reducing the interference of external pressure on marital decisions. According to conservation of resources theory (Hobfoll, 1989), individuals who believe they possess sustainable psychological resources are more likely to make commitments and decisions. Thus, psychological capital enhances individuals’ recognition of the value of marriage and their confidence in the relationship, thereby increasing their willingness to marry.

Based on the above analysis, this study proposes the following hypothesis:

*H3*: Psychological capital is positively correlated with marital intention.

Psychological capital shapes individuals’ assessments and decision-making in the process of resource exchange. As a key internal positive psychological resource, when individuals face the threat of resource loss, psychological capital exerts an indirect effect through either its depletion or its activation. The depletion of psychological capital affects marital intention via two pathways. First, individuals with low psychological capital are more likely to perceive marriage as a “resource-exhausting exchange,” believing that they lack sufficient positive psychological resources to cope with the uncertainties inherent in marriage, thereby raising their threshold for assessing marital benefits. Second, insufficient psychological capital exacerbates the negative emotional cycle triggered by economic pressure; the absence of positive psychological states amplifies anxiety. According to Hobfoll’s (1989) conservation of resources theory, when individuals face the depletion of valued resources, they tend to adopt resource conservation strategies—namely, avoidance behaviors. In the context of this study, when an individual’s psychological resources are excessively depleted due to economic pressure, they are likely to avoid entering a marital relationship that requires further resource investment.

Based on the above analysis, this study proposes the following hypothesis:

*H4*: Psychological capital mediates the relationship between economic pressure and marital intention.

### 2.3 Research model and hypothesis summary

**Figure 1:**
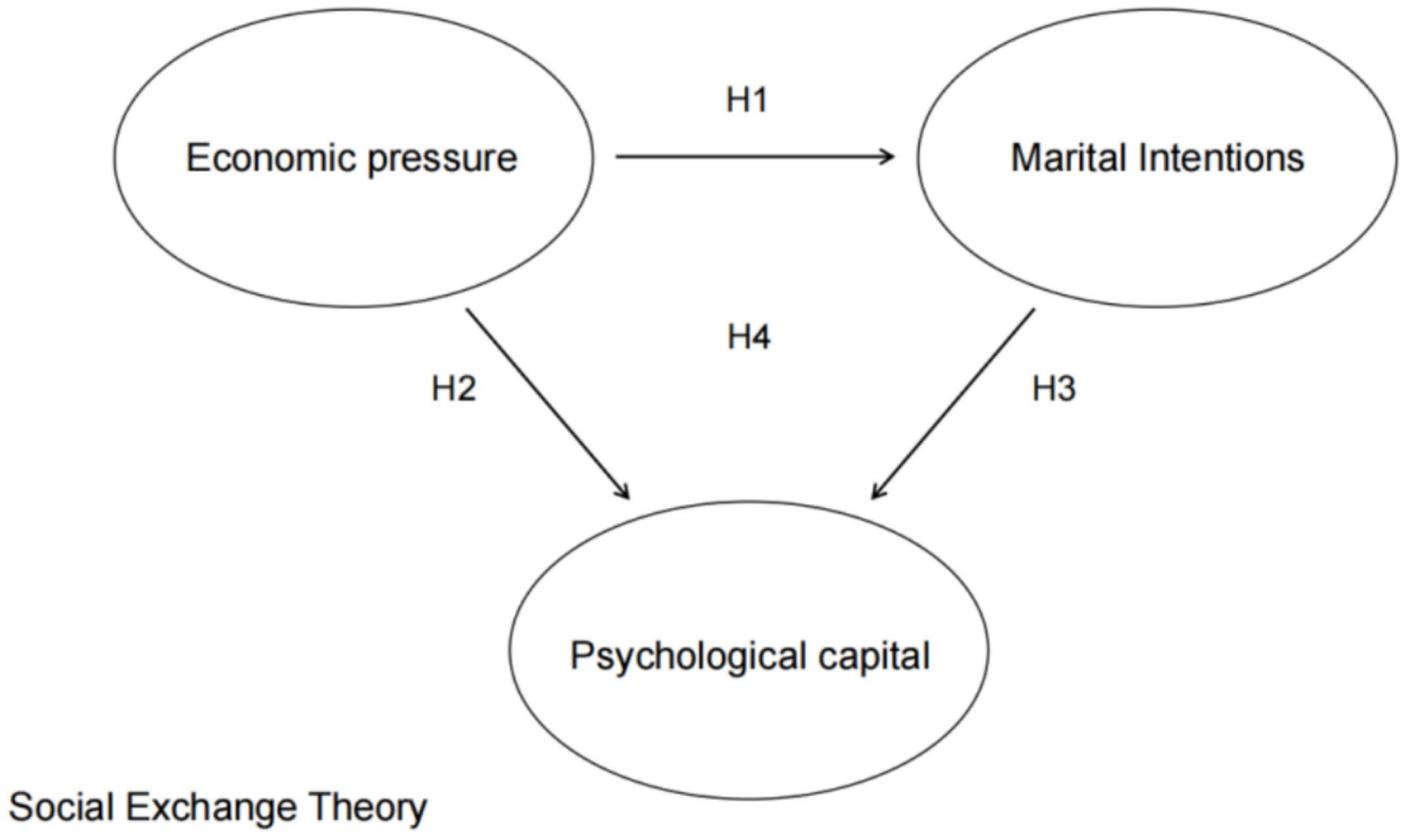
Theoretical model.

This study focuses on young people aged 20 to 35. Drawing on social exchange theory and employing psychological capital as a mediating variable, it analyzes the internal influence mechanism and transmission pathway through which economic pressure affects their marital intentions. This chapter elaborates on the theoretical basis for model construction and the specific research hypotheses. The proposed research model is presented in Figure 1.

Drawing on the research model derived from social exchange theory, this study formulates the following hypotheses concerning the relationships between economic pressure, psychological capital, and marital intention (see Table 1).

**Table 1:** Hypothesis summary.

Serial number	Hypotheses
H1	Economic pressure is negatively associated with marital intentions.
H2	Economic pressure has a significant negative impact on psychological capital.
H3	Psychological capital is positively correlated with marital intention.
H4	Psychological capital mediates the relationship between economic pressure and marital intention.

## 3 Research method

### 3.1 Data sources

This study employed a questionnaire survey method. Taking into account the life cycle of education, employment, and marriage among Chinese youth, as well as China’s legal regulations on the minimum marriage age (22 for men and 20 for women), the target population was defined as unmarried Chinese young people aged 20 to 35. In this study, “unmarried” specifically refers to individuals who have never had a legal marriage registration, encompassing various emotional states such as being single or in a relationship. This definition allows the study to focus on their willingness to transition from being unmarried to entering a first marriage and the factors influencing that transition.

The survey was conducted in four cities in the Beijing-Tianjin-Hebei region: Beijing, Tianjin, Shijiazhuang, and Baoding. As the most representative urban agglomeration in northern China, the Beijing-Tianjin-Hebei region has a dense population, and its residents’ views on marriage and childbearing are significantly influenced by policies, providing valuable insight into the overall marriage and childbearing landscape in China. The four selected cities exhibit a notable income gradient: Beijing is a high-income first-tier city; Tianjin is a coastal city positioned between the first and second tiers; Shijiazhuang is a second-tier city; and Baoding is a third-tier city with relatively lagging economic development.

This study collected data through quantitative questionnaires covering economic pressure, psychological capital, and marital intention, and preliminarily examined the relationships among these variables. To reduce common method bias, data were collected in two waves, separated by a two-week interval. The first wave measured demographic and explanatory variables, while the second wave measured mediating and outcome variables. This temporal separation helped mitigate biases caused by habitual response patterns and improved the authenticity and accuracy of the responses. In the first wave, 225 electronic questionnaires were distributed, and 215 valid responses were received, yielding a valid response rate of 95.56%. Two weeks later, a second wave was conducted, with 220 questionnaires distributed and 210 valid responses received, resulting in a valid response rate of 95.45%. In total, 425 valid questionnaires were collected for this study.

### 3.2 Measures

Regarding the three core variables—economic pressure, psychological capital, and marital intention—this study prioritized the effectiveness of scale localization in the Chinese context. Ultimately, scales published in authoritative academic journals were selected, and semantic equivalence between the Chinese and English versions was ensured through a translation and back-translation process prior to formal use. This procedure guaranteed the scientific rigor and cultural adaptability of the measurement tools.

As the selected scales were originally developed in English, this study strictly followed the translation-back-translation procedure proposed by Brislin (1970) to ensure semantic, conceptual, and cultural equivalence between the Chinese and original versions. Two master’s students in psychology independently translated the English scale into Chinese, producing two draft translations. These drafts were compared and discussed to develop an initial Chinese version. Subsequently, two graduate students majoring in English independently back-translated the initial Chinese version into English. The back-translated versions were then compared with the original scale item by item to identify semantic discrepancies, and the Chinese expressions were revised accordingly to finalize the scale.

To accurately estimate the net effect of economic pressure on young people’s marital intentions, this study controlled for several variables in the model, including sex, age, educational level, political affiliation, and personal monthly income. Existing research indicates significant sex differences in the perception of marriage costs and expected benefits. Age, meanwhile, reflects the life cycle stage at which marriage decisions are made, with young people of different ages facing varying social expectations and physiological pressures. In the Chinese context, political affiliation may serve as a proxy for an individual’s social networks and values, which could indirectly influence their marital attitudes and decisions. Finally, educational level and personal monthly income, as indicators of objective socioeconomic status, help disentangle the subjective perception of economic pressure from objective resource conditions, thereby allowing for a more precise identification of the effect of economic pressure itself on marital intentions. As key sociodemographic characteristics, these variables may independently influence individuals’ marital decisions. Controlling for them helps rule out alternative explanations, thereby ensuring a more robust estimation of the core variable relationships and the mediating role of psychological capital within the framework of social exchange theory.

The explanatory variable in this study is economic pressure, defined as a negative psychological state that young people experience when they perceive their available resources as insufficient to meet current and anticipated living needs (Drentea and Reynolds, 2015). This study draws on the family stress model proposed by Conger et al. (1994) and examines the variable from the perspective of perceived economic pressure. To measure economic pressure, this study employs the Perceived Economic Scarcity Scale developed by Auger et al. (2024). Sample items include “My income seems very limited compared to others” and “I am worried that the assets I hold are not enough” (Auger et al., 2024). A 5-point Likert scale was adopted, with higher scores indicating greater economic pressure.

The mediating variable in this study is psychological capital, defined as a positive psychological state exhibited by individuals during their growth and development. Consistent with the research focus, this study employed the 24-item Psychological Capital Questionnaire (PCQ-24) developed by Luthans et al. (2007). This scale has demonstrated good reliability and validity in prior research (Luthans et al., 2007) and uses a 5-point Likert scoring format. The PCQ-24 has been translated into multiple languages and has shown strong reliability and validity in studies conducted within the Chinese context. Its four dimensions—self-efficacy, hope, optimism, and resilience—enable a comprehensive examination of how economic pressure depletes different psychological traits in individuals, thereby facilitating further exploration of the mediating pathways of psychological capital.

The dependent variable in this study is marital intention, which reflects an individual’s subjective tendency and perceived likelihood of entering marriage within a certain future timeframe. This subjective tendency represents the individual’s evaluation of and personal endorsement of the value of marriage, and is considered a relatively stable psychological disposition (Li et al., 2025).To measure marital intention, this study employed the positive items from the Marital Intention Scale (IMS) and the General Marital Attitude Scale (GAMS) developed by Park and Rosén (2013). This scale has been widely used in recent domestic studies on youth marriage and romantic relationships and has demonstrated good applicability. To avoid potential misunderstandings associated with reverse-scored items and to enhance the accuracy of data collection, only the positive items were selected, including statements such as “I want to get married” (Park and Rosén, 2013). A 5-point Likert scale was adopted, with higher scores indicating stronger marital intention. The scale was adaptively modified for the context of this study, resulting in the final questionnaire.

This study used Mplus 8.0 structural equation modeling and SPSS 26.0 to analyze questionnaire data. Specifically, descriptive analysis was conducted on control variables such as sex, age, and education using SPSS, and differential analysis and reliability testing were performed on each variable; Mplus was used to compare the validity of various variables through model competition, and the relevant hypotheses of this study were also validated.

## 4 Results

### 4.1 Descriptive statistics

Among the 425 questionnaires, there were 247 females, accounting for 58.12%; and 178 males, accounting for 41.88%. In terms of age distribution, there were 98 people aged 20–22, accounting for 23.06%; 132 people aged 23–25, accounting for 31.06%; 138 people aged 26–30, accounting for 32.47%; and 57 people aged 31–35, accounting for 13.41%. Regarding educational background, there were 62 people with a high school education or below, accounting for 14.59%; 274 people with a college or undergraduate degree, accounting for 64.47%; and 89 people with a master’s degree or above, accounting for 20.94%. Regarding the political affiliation of the respondents, there were 89 Communist Party members, accounting for 20.94%; 64 probationary Communist Party members, accounting for 15.06%; 37 Communist Youth League members, accounting for 8.71%; and 235 ordinary people, accounting for 55.29%. In terms of the respondents’ personal monthly income, there were 79 people with an income of 3,000 yuan or less, accounting for 18.59%; 133 people with an income of 3,001–6,000 yuan, accounting for 31.29%; 51 people with an income of 6,001–10,000 yuan, accounting for 12%; 132 people with an income of 10,001–15,000 yuan, accounting for 31.06%; and 30 people with an income of 15,000 yuan or more, accounting for 7.06% (see Table 2).

**Table 2:** Demographic description.

Name	Options	Frequency	Percentage (%)
Sex	Female	247	58.12
	Male	178	41.88
Age	20–22 years old	98	23.06
	23–25 years old	132	31.06
	26–30 years old	138	32.47
	31–35 years old	57	13.41
Education	High school and below	62	14.59
	Junior college and undergraduate	274	64.47
	Master’s degree and above	89	20.94
Political affiliation	Communist Party member	89	20.94
	Prospective Communist Party member	64	15.06
	Member of the Communist Youth League	37	8.71
	The masses	235	55.29
Personal monthly income	3,000 yuan and below	79	18.59
	3,001–6,000 yuan	133	31.29
	6,001–10,000 yuan	51	12
	10,001–15,000 yuan	132	31.06
	15,000 yuan and above	30	7.06
Total		425	100

### 4.2 Relevance

According to Table 3, economic pressure shows a significant negative correlation with psychological capital and marital intention, while psychological capital has a positive impact on marital intention.

**Table 3:** Descriptive statistics and correlation analysis.

Variable	1	2	3	4	5	6	7	8
1. Sex	1							
2. Age	0.066	1						
3. Education	0.027	−0.015	1					
4. Political affiliation	−0.027	0.094	0.056	1				
5. Personal monthly income	0.117*	−0.006	−0.119*	−0.025	1			
6. Economic pressure	−0.107*	−0.059	−0.003	−0.029	−0.021	1		
7. Psychological capital	0.116*	0.019	−0.042	0.013	0.013	−0.392**	1	
8. Marital intention	0.118*	0.083	0.051	−0.016	0.022	−0.411**	0.529**	1
Mean	1.58	2.36	2.06	2.98	3.03	2.87	3.08	2.99
SD	0.494	0.981	0.593	1.243	1.123	1.079	0.851	0.881

*n* = 425, *p < 0.05, **p < 0.01. Sex: 1 = Female, 2 = Male/Age: 1 = 20 years old to 22 years old, 2 = 23 years old to 25 years old, 3 = 26 years old to 30 years old, 4 = 31 years old to 35 years old/Education: 1 = High school education or below, 2 = Master’s degree or above/ Political Affiliations:1 = Member of the Communist Party of China, 2 = Preparatory member of the Communist Party of China, 3 = League member,4 = Common Citizen/Love State:1 = YES,2 = NO/Personal monthly income: 1 = 3,000 yuan and below, 2 = 3,001–6000yuan, 3 = 6,001–10000yuan, 4 = 10,001–15000yuan, 5 = Above 15,001 yuan.

### 4.3 Difference analysis

This paper uses the independent samples *t*-test to study the differences in sex on three aspects: economic pressure (EP), psychological capital (PC), and marital intention (MI). The data results (Table 4) show that there is no significant difference in EP and MI between sexes (*p* > 0.05). However, there is a significant difference in PC (*p* < 0.05), indicating that different sex samples have differences in PC.

**Table 4:** Analysis of sex differences in different variables.

Variable	Sex (mean ± standard deviation)		*t*	*p*
	Male (*n* = 178)	Female (*n* = 247)		
EP	3.08 ± 0.92	2.98 ± 0.90	−1.12	0.263
PC	3.07 ± 0.86	3.27 ± 0.79	−2.524	0.012*
MI	2.89 ± 0.82	2.79 ± 0.79	−1.367	0.172

*p < 0.05.

The different sex samples show a significant difference in PC (*t* = −2.492, *p* = 0.012). The scores of male samples are lower than those of female samples (3.07 < 3.27), which also indicates that compared to men, women tend to have a more positive psychological state regarding marriage. Women have higher psychological capital in marriage than men. This can be analyzed from three aspects. First, in China, from a sex perspective, women tend to exhibit stronger compliance with norms and social adaptability during their growth. This trait makes them more likely to accept social norms and family education, and when it comes to marriage, they demonstrate a higher level of psychological capital. Second, at the same age, women’s emotional cognition and social development often precede those of male peers. Third, the imbalance in the ratio of men to women increases the psychological pressure on men.

As shown in Table 5, there were no significant differences among different age groups in terms of economic pressure (EP), psychological capital (PC), and marital intention (MI) (*p* > 0.05).

**Table 5:** Analysis of differences in age across different variables.

Variable	Age (mean ± standard deviation)				*F*	*p*
	20–22 years old (*n* = 98)	23–25 years old (*n* = 132)	26–30 years old (*n* = 138)	31–35 years old (*n* = 57)		
EP	2.98 ± 0.98	3.09 ± 0.84	2.98 ± 0.94	3.13 ± 0.91	0.644	0.587
PC	3.13 ± 0.88	3.20 ± 0.80	3.19 ± 0.84	3.21 ± 0.77	0.178	0.912
MI	2.84 ± 0.79	2.77 ± 0.77	2.85 ± 0.86	3.04 ± 0.76	1.463	0.224

As shown in Table 6, there were no significant differences in education level among the three factors: economic pressure (EP), psychological capital (PC), and marital intention (MI) (*p* > 0.05).

**Table 6:** Analysis of differences in education level across different variables.

Variable	Education (mean ± standard deviation)			*F*	*p*
	High school and below (*n* = 62)	Junior college and undergraduate levels (*n* = 274)	Master’s degree or above (*n* = 89)		
ER	2.99 ± 0.89	3.00 ± 0.93	3.18 ± 0.87	1.443	0.237
PC	3.35 ± 0.76	3.14 ± 0.85	3.22 ± 0.78	1.774	0.171
MI	2.81 ± 0.87	2.85 ± 0.80	2.86 ± 0.79	0.089	0.915

The data results (Table 7) show that there were no significant differences in political affiliation among the groups in terms of economic pressure (EP), psychological capital (PC), and marital intention (MI) (*p* > 0.05).

**Table 7:** Analysis of differences in political affiliation across different variables.

Variable	Political affiliation (mean ± standard deviation)				*F*	*p*
	Communist Party member (*n =* 89)	A probationary member of the Communist Party of China (*n* = 64)	Member of the Communist Youth league (*n* = 37)	The masses (*n* = 235)		
EP	3.04 ± 0.89	2.82 ± 0.98	2.81 ± 0.90	3.13 ± 0.90	1.004	0.405
PC	3.20 ± 0.82	3.18 ± 0.81	2.81 ± 0.90	3.24 ± 0.81	1.223	0.300
MI	2.97 ± 0.86	2.76 ± 0.75	2.69 ± 0.66	2.85 ± 0.81	0.840	0.501

As shown in Table 8, there was no significant difference in economic pressure (EP) among individuals (*p* > 0.05), but significant differences were observed in psychological capital (*p* = 0.047) and marital intention (*p* = 0.033). Overall, the higher an individual’s monthly income, the more positive psychological state they showed towards marriage, and the stronger their marital intention was.

**Table 8:** Analysis of differences in personal monthly income across different variables.

Variable	Personal monthly income (mean ± standard deviation)					*F*	*p*
	3,000 yuan and below (*n* = 79)	3,001–6,000 yuan (*n* = 133)	6,001–10,000 yuan (*n* = 51)	10,001–15,000 yuan (*n* = 132)	15,000 yuan and above (*n* = 30)		
EP	2.92 ± 0.96	3.07 ± 00.85	3.22 ± 0.95	3.02 ± 0.93	2.93 ± 0.95	1.460	0.225
PC	3.11 ± 0.81	3.11 ± 0.86	3.21 ± 0.89	3.23 ± 0.80	3.37 ± 0.74	2.676	0.047*
MI	2.81 ± 0.83	2.66 ± 0.93	2.83 ± 0.76	2.93 ± 0.74	2.91 ± 0.80	2.936	0.033*

*p < 0.05.

### 4.4 Reliability testing

Reliability testing, also known as reliability analysis, refers to the consistency, stability, and reliability of the measured data. Generally, internal consistency is used to indicate the reliability of the test, mainly by calculating the Cronbach’s alpha coefficient of the scale to evaluate the reliability of the questionnaire. The larger the Cronbach’s alpha coefficient, the stronger the consistency between variables in the scale and the higher the reliability of the scale. When Cronbach’s alpha is greater than 0.7, it indicates that the variable has good internal consistency. As shown in Table 9, the reliability of each variable involved in this study was 0.941, 0.971, and 0.934, respectively, and the Cronbach’s alpha of each variable was greater than 0.7. This indicates that the scale and questionnaire used in this study have good internal consistency, and the analysis results have high reliability (see Table 9).

**Table 9:** Reliability analysis of various variables.

Variable	Cronbach’s alpha	Number of question items
Economic pressure	0.941	9
Psychological capital	0.971	24
Marital intention	0.934	11

### 4.5 Confirmatory factor analysis

This study conducted confirmatory factor analysis using Mplus 8.0 software to test the hypothesis model by substituting actual data into it, in order to verify whether the theoretical model is consistent with the observed data. Specifically, it is divided into benchmark model, two factor model, and single factor model. The fitness index includes commonly used statistical measures such as chi square test value, RMSEA, CFI, TLI, etc., supplemented by factor loading significance test to confirm the explanatory power and reliability of each variable’s corresponding factor. Through this test, the structural validity and predictive power of the model are further enhanced. As shown in Table 10, it was found that there were certain issues with the indicators of the two factor and single factor models in the model, and there was a certain mismatch between the model and the data. To determine the most suitable benchmark model for interpreting observational data, the model data has good structural validity.

**Table 10:** Confirmatory factor analysis.

Model	*χ*^2^	df	*χ*^2^/df	CFI	TLI	RMSEA	SRMR
Baseline Model (EP; PC; MI)	2112.288	1,214	1.74	0.940	0.937	0.042	0.064
Two Factor Model (EP; PC + MI)	11915.102	1,260	9.46	0.293	0.285	0.141	0.302
Single Factor Model (EP + PC + MI)	14510.872	1,268	11.44	0.122	0.117	0.157	0.315

*N* = 425, EP, economic pressure; PC, Psychological Capital; MI, marital intention; CFI, Comparative Fit Index; TLI, Tucker Lewis index; RMSEA, Root Mean Square Error Approximate Value; SRMR, Standardized Root Mean Square Residual.

Evaluate the benchmark model, which includes three variables: economic pressure, psychological capital, and marital intention. Its chi square/df value is 1.74 less than 3, CFI is 0.940 greater than 0.9, TLI is 0.937 greater than 0.9, RMSEA is 0.042 less than 0.08, and SRMR is 0.064 less than 0.08. The CFA indicator shows that the benchmark model performs well in all fitting indicators, indicating that it is most suitable for interpreting observed data and has good data structure validity. The data has been verified through confirmatory factor analysis, and the fitting state of the data is good.

The two factor model consists of two variables: economic pressure, psychological capital, and marital intention, with a chi square/df value of 9.46, which is greater than 3. The CFI is 0.293 and the TLI is 0.285, both less than 0.8, indicating poor goodness of fit for the two factor model. RMSEA is 0.141 and SRMR is 0.302, both of which are greater than 0.08, indicating that the two factor model has a poorer fitting effect compared to the baseline model.

The single factor model is a combination of three variables, with a chi square/df value of 11.44, CFI of 0.122, TLI of 0.117, RMSEA of 0.157, and SRMR of 0.315. There are certain issues with each indicator, namely the poor fitting effect of the single factor model compared to the benchmark model.

Through comprehensive comparison, it is found that the benchmark model has the best fitting effect, indicating that the current data has a certain good structural validity.

### 4.6 Common method deviation testing

This article uses the Harman single factor analysis method to test the items of the three scales in this study on the collected data. The results showed that the variance explanation rate of the first factor was only 27.219%, which did not reach the critical value. That is, the impact of common method bias on the research data was within a reasonable range, and there was no serious problem of common method bias (see Table 11).

**Table 11:** Common method deviation.

Ingredient	Initial eigenvalue			Extract the sum of squared loads		
	Total	Variance percentage	Accumulated%	Total	Variance percentage	Accumulated%
1	17.693	27.219	27.219	17.693	27.219	27.219
…	…	…	…	…	…	…
65	0.072	0.111	100			

### 4.7 Analysis of the effect of economic pressure on marital intention

This study hypothesizes that economic pressure has a significant negative impact on marital intention (Hypothesis 1). To verify this hypothesis, this study used the Mplus structural equation model to analyze the collected data on economic pressure and marital intention. The results showed (as shown in Table 12) that there was a significant negative correlation between economic pressure and marital intention (*β* = −0.411, *p* < 0.01), which means that the greater the economic pressure, the lower the marital intention. Conversely, the smaller the economic pressure, the stronger the marital intention of young people. It can be seen that the economy is an important factor affecting people’s willingness to marry. Therefore, hypothesis 1 of this study has been validated, and it is confirmed that economic pressure has a significant negative impact on marital intention.

**Table 12:** Analysis of economic pressure and marital intention results.

Variable	Marital intention			
	Estimate	S.E.	Est./S.E.	*p*
Economic Pressure	−0.411**	0.040	−9.214	0.000

**p < 0.01.

### 4.8 Analysis of the effect of economic pressure on psychological capital

This study assumes that economic pressure has a significant negative impact on psychological capital (Assumption 2). To verify this hypothesis, this study used the Mplus structural equation model to analyze the collected data on economic pressure and psychological capital. Research has shown (as shown in Table 13) that economic pressure has a significant negative impact on psychological capital (*β* = −0.392, *p* < 0.01), meaning that the greater the economic pressure, the lower the psychological capital of young people; the smaller the economic pressure, the higher the psychological capital. This means that economic pressure affects the intensity of young people’s positive psychological state towards marriage. Therefore, hypothesis 2 of this study has been validated, and economic pressure has a significant negative impact on psychological capital.

**Table 13:** Analysis of economic pressure and psychological capital results.

Variable	Psychological capital			
	Estimate	S.E.	Est./S.E.	*p*
Economic pressure	−0.392**	0.043	−9.039	0.000

**p < 0.01.

### 4.9 Analysis of the effect of psychological capital and marital intention

This study suggests a positive correlation between psychological capital and marital intention (Assumption 3). To verify this hypothesis, this study analyzed it using the Mplus structural equation model. Research has shown (as shown in Table 14) that psychological capital has a significant positive impact on marital intention (*β* = 0.529, *p* < 0.01), meaning that the higher the psychological capital, the stronger the marital intention of young people; the lower the psychological capital, the weaker the willingness to marry. Therefore, hypothesis 3 of this study has been validated, and the positive correlation between psychological capital and marital intention is established.

**Table 14:** Analysis of psychological capital and marital intention results.

Variable	Marital intention			
	Estimate	S.E.	Est./S.E.	*p*
Psychological Capital	0.529**	0.046	11.615	0.000

**p < 0.01.

### 4.10 Analysis of the mediating effect of psychological capital on economic pressure and marital intention

This study assumes that economic pressure will ultimately affect young people’s willingness to marry through the mediating transmission mechanism of psychological capital (Hypothesis 4). There are two types of mediation effects: complete mediation and partial mediation. Complete mediation refers to the fact that the influence of the argument on the dependent variable is entirely achieved through the mediator variable, that is, after adding the mediator variable, the direct effect of the independent variable on the dependent variable is not significant, and the influence of the independent variable on the dependent variable depends entirely on the role of the mediator variable. When the direct effect is not significant but the indirect effect is significant, the mediation result is a complete mediation effect; Partial mediation refers to the transmission of the influence of arguments on the dependent variable through the mediator variable. That is, after adding the mediator variable, the direct effect of the independent variable on the dependent variable remains significant. The independent variable not only indirectly affects the dependent variable through the mediator variable, but also directly affects the dependent variable. If both direct and indirect effects are significant, then the mediation result is a partial mediation effect.

In order to verify the mediating role of psychological capital between economic pressure and marital intention, this study explored the relationships between economic pressure and marital intention, economic pressure and psychological capital, and psychological capital and marital intention.

The research results (as shown in Table 15) indicate that in the path of “economic pressure → marital intention”, the path is denoted as a, with a direct effect coefficient of −0.185 (*p* < 0.01), a standard error of 0.040, and a 97.5% BootCI confidence interval of (−0.561, −0.413).

**Table 15:** Mediation effect test.

Item	*β*	S.E.	*p*	97.5% BootCI	
Economic Pressure → Marital Intention	−0.185**	0.040	0.000	−0.561	−0.413
Economic Pressure →Psychological Capital	−0.483**	0.039	0.000	−0.261	−0.109
Psychological Capital → Marital Intention	0.571**	0.040	0.000	0.494	0.653
Economic Pressure → Psychological Capital → Marital Intention	−0.276**(Direct Effect)	0.030	0.000	−0.339	−0.222
Economic Pressure → Psychological Capital → Marital Intention	0.598**(Indirect Effect)	0.068	0.000	0.473	0.748

**p < 0.01.

In the path of “economic pressure → psychological capital”, the path is denoted as b, with a direct effect coefficient of −0.483 (*p* < 0.01), a standard error of 0.039, and a 97.5% BootCI confidence interval of (−0.261, −0.109).

In the path of “psychological capital → marital intention”, denoted as c, its direct effect coefficient is 0.571 (*p* < 0.01), standard error is 0.040, and 97.5% BootCI confidence interval is 0.494–0.653.

In the intermediary path of “economic pressure → psychological capital → marital intention”, the path is a * b, with a direct effect value of −0.276 (*p* < 0.01), a standard error of 0.030, and a 97.5% BootCI confidence interval of (−0.339, −0.222), which does not include 0, indicating significant direct effect. The indirect effect is 0.598 (*p* < 0.01), the standard deviation is 0.068, and the 97.5% BootCI confidence interval is 0.473–0.748, which does not include 0, indicating significant indirect effects. In summary, both direct and indirect effects are significant, and this mediating pathway is established and partially mediated. Thus, hypothesis 4 is validated, that is, psychological capital plays a mediating role in the relationship between economic pressure and marital intention.

## 5 Summary and discussion

### 5.1 Hypothesis testing summary

This study is based on the Mplus structural equation model and systematically integrates relevant literature such as economic pressure, marital intention, and psychological capital. Based on existing research, this article further explores how economic pressure affects young people’s willingness to marry by introducing psychological capital as a mediating variable. The statistical analysis data comes from 425 valid questionnaires obtained from a questionnaire survey. After preprocessing and analyzing the data, hypothesis relationships between variables are tested. The specific statistical analysis results are shown in Table 16.

**Table 16:** Summary of hypothesis testing.

Serial number	Assuming content	Verification result
H1	Economic pressure is negatively associated with marital intentions.	Established
H2	Economic pressure has a significant negative impact on psychological capital.	Established
H3	Psychological capital is positively correlated with marital intention.	Established
H4	Psychological capital mediates the relationship between economic pressure and marital intention.	Established

The statistical analysis results are shown in the table above, and all research hypotheses are valid.

### 5.2 Summary of research conclusions

By studying the impact of economic pressure on young people’s willingness to marry, this article has drawn some conclusions with theoretical significance and practical value.

1. The economic pressure on young people has a significant negative impact on their willingness to marry. This conclusion also conforms to the theoretical inference of social exchange relationships, that is, the maintenance of a relationship depends on the balance between rewards and costs (Liu et al., 2025). Economic pressure can significantly increase the costs of marriage and reduce the sense of security and quality of life benefits. When the perceived costs exceed the benefits, the willingness to enter or maintain marriage will decrease.
2. Economic pressure has a significant negative impact on psychological capital. This conclusion is consistent with the conclusion proposed by Nasir et al. (2025) that high financial pressure leads to a significant increase in the risk of psychological distress. Suleman and Carvalho (2024) also proposed in his research that when graduates are filled with anxiety and uncertainty about the future, they may experience psychological symptoms such as depression and anxiety.
3. There is a positive correlation between psychological capital and young people’s willingness to marry. This conclusion is consistent with current research, as both domestic and international studies on the relationship between psychological capital and youth romantic relationships (Zhang et al., 2015) and marital quality (Hoseiny et al., 2019) have shown a positive correlation between psychological capital and various aspects of youth marital intentions.
4. Psychological capital plays a mediating role in the influence of economic pressure on marital intentions. This conclusion provides a new perspective for understanding the impact of economic pressure on marriage intention and analyzing the relationship between the two.

### 5.3 Mechanism analysis and summary

From the perspective of social exchange theory, marriage is regarded as a social exchange behavior that involves investing resources and expecting returns. Traditional explanations have focused on the material aspect—that is, the greater the economic pressure, the more difficult it is for individuals to meet the material thresholds of marriage, and thus their marital intentions decrease. This study made a rough estimation based on the data released by the National Bureau of Statistics. In the four cities of Beijing, Tianjin, Shijiazhuang and Baoding, the annual average living costs for a married family (with one child) in the cities were 152,691 yuan, 112,758 yuan, 94,000 yuan and 80,000 yuan respectively, and the annual minimum living costs also reached 60,300 yuan, 44,700 yuan, 34,900 yuan and 31,900 yuan, respectively.

However, existing research shows that the influence of economic pressure on marital intentions is not limited to the material dimension; it can also affect marital decisions by triggering emotional responses (Tian et al., 2019). This study argues that the formation of marital intentions depends not only on “whether one can bear the cost of marriage,” but also on “whether an individual has sufficient psychological resources to perceive and cope with the uncertainties inherent in marriage.” Psychological capital, as a mediating variable, primarily functions through the following mechanisms.

First, psychological capital affects an individual’s cognition and assessment of marriage. Unmarried young people’s evaluations of marriage are shaped by their psychological expectations, which are closely related to their psychological resources (Li et al., 2025). Individuals with high psychological capital tend to view marriage as a positive cooperative endeavor, with higher expected gains and more flexible mate selection criteria, enabling them to maintain strong marital intentions even under economic pressure. In contrast, individuals with low psychological capital are more likely to perceive marriage as a drain on resources, exaggerating its costs and thereby reducing their marital intentions.

Second, psychological capital influences an individual’s emotional state during marital decision-making. Insufficient economic resources often trigger negative emotions such as anxiety and depression, which can interfere with marital judgments. As a positive emotional resource, psychological capital can effectively buffer these negative emotions and mitigate the interference of external pressure on decision-making. Individuals with low psychological capital, however, are more susceptible to being dominated by negative emotions and tend to adopt avoidance behaviors as a means of preserving resources.

Third, psychological capital affects an individual’s tendency toward resource preservation and investment. According to conservation of resources theory, when individuals face the threat of resource loss, they tend to reduce external investments (Hobfoll, 1989). Marriage, as a relationship requiring long-term commitment, is essentially a form of “resource investment behavior.” Individuals with high psychological capital are more willing to invest resources under conditions of uncertainty because they believe in their ability to cope with future challenges. In contrast, those with low psychological capital are more inclined to adopt a “resource conservation” strategy, viewing marriage as a high-risk, low-return endeavor, which ultimately diminishes their marital intentions.

### 5.4 Research limitations and prospects

Although this study initially revealed the mechanism by which economic pressure influences young people’s marital intentions through psychological capital, several limitations should be acknowledged and addressed in future research.

First, the cross-sectional nature of the research design presents limitations. All variables were measured at a single time point, which can only explain covariation among variables and cannot establish temporal sequences or dynamic evolution. This raises the possibility of reverse causality—for instance, while economic pressure may weaken psychological capital and reduce marital intentions, individuals with lower marital intentions might also exaggerate economic hardships to rationalize their attitudes. Moreover, the effects of economic pressure and psychological capital may involve long-term cumulative effects or stage-specific fluctuations. Cremers et al. (2024) noted that ignoring time-varying endogenous covariates in longitudinal studies can lead to estimation bias, and that joint models can provide more robust causal inferences. Future research should adopt longitudinal designs to track the long-term impact of these variables on marital intentions over time.

Second, the regional limitations and structural imbalance of the sample affect the generalizability of the findings. This study primarily selected unmarried young people from Beijing, Tianjin, Shijiazhuang, and Baoding—cities concentrated in northern China—and thus failed to fully capture China’s regional heterogeneity, including significant differences in economic development, housing policies, and marriage norms, which may influence the strength of the effect of economic pressure on marital intentions. Mao et al. (2024), drawing on national data from 32,282 respondents, found that while urban–rural and regional differences exist, attitudes toward marriage and childbearing show a consistent overall trend, with women exhibiting decreased tolerance for unsatisfactory marriages and more negative attitudes toward marriage and childbearing. This suggests that interaction effects between sex and region may not have been fully captured in the present study. Xu (2020) emphasized the importance of family and educational background in shaping marriage and romantic relationships. Future research should employ multi-stage stratified sampling nationwide to include samples from diverse economic regions.

Third, the cultural adaptability of the measurement tools affects the accuracy of construct measurement. Although the scales used in this study are widely applied, some items may not fully align with Chinese young people’s understandings of economic pressure and marriage within the local cultural context. If these scales fail to capture localized connotations, this may lead to underestimation of the effects of economic pressure or introduce measurement bias. Shackelford (2022) noted that cross-cultural research must address methodological issues ranging from concept definition to scale equivalence. Future research should integrate qualitative interviews and other methods to develop or revise measurement tools that are better suited to the Chinese context, thereby enhancing the cultural sensitivity and ecological validity of the research.

Fourth, the simplicity of the model construction may lead to a one-sided understanding of the underlying mechanisms. Although this study controlled for variables such as sex, age, and income, it did not include important factors such as family support, social norms, marital attitudes, housing pressure, or media influence. Jang and Cho (2025) found that social comparison and materialism affect marital intentions through the moderating role of anxiety, revealing complex psychological mechanisms; marriage payment customs such as betrothal gifts also amplify economic pressure. Chae and Zhang (2026) found that men in regions with high betrothal gifts have significantly lower expectations of marrying and having children before age 30, suggesting that economic burdens may delay family formation and career development. The omission of these variables may result in an incomplete understanding of the mechanisms at play. Future research should expand the theoretical framework to include more sociocultural and environmental variables and examine the heterogeneity of these mechanisms across different groups, in order to develop a more comprehensive understanding of the formation of young people’s marital intentions.

## Data Availability

The datasets presented in this article are not readily available because 30% of the respondents in this study are current master’s students and public officials who refuse to disclose information due to their professions and future career aspirations. Requests to access the datasets should be directed to LJ, liushuhan1980@163.com.

## References

[ref1] Auger V. Sommet N. Normand A. (2024). The perceived economic scarcity scale: a valid tool with greater predictive utility than income. Br. J. Soc. Psychol. 63, 1112–1136. doi: 10.1111/bjso.12719, 38205924

[ref2] Avey J. B. Avolio B. J. Crossley C. D. Luthans F. (2009). Psychological ownership: theoretical extensions, measurement and relation to work outcomes. J. Organ. Behav. 30, 173–191. doi: 10.1002/job.583

[ref3] Brislin R. W. (1970). Back-translation for cross-cultural research. J. Cross-Cult. Psychol. 1, 185–216. doi: 10.1177/135910457000100301

[ref4] Chae M. Zhang D. (2026). Bride price, marriage expectations, and intentions among young male migrants in China: evidence from manufacturing gig workers. Econ. Model. 155:107443. doi: 10.1016/j.econmod.2025.107443

[ref5] Conger R. D. Donnellan M. B. (2007). An interactionist perspective on the socioeconomic context of human development. Annu. Rev. Psychol. 58, 175–199. 16903807 10.1146/annurev.psych.58.110405.085551

[ref6] Conger R. D. Ge X. Elder G. H. Jr. Lorenz F. O. Simons R. L. (1994). Economic stress, coercive family process, and developmental problems of adolescents. Child Dev. 65, 541–561. 8013239

[ref7] Cremers J. Mortensen H. L. Ekstrøm T. C. (2024). A joint model for longitudinal and time-to-event data in social and life course research: employment status and time to retirement. Sociol. Methods Res. 53, 603–638. doi: 10.1177/00491241211055770

[ref8] Drentea P. Reynolds R. J. (2015). Where does debt fit in the stress process model? Soc. Ment. Health 5, 16–32. doi: 10.1177/2156869314554486 31106006 PMC6521877

[ref9] Elahi A. McIntyre J. C. Hampson C. Bodycote H. J. Sitko K. White R. G. . (2018). Home is where you hang your hat: host town identity, but not hometown identity, protects against mental health sympto ms associated with financial stress. J. Soc. Clin. Psychol. 37, 159–181. doi: 10.1521/jscp.2018.37.3.159

[ref10] Hobfoll S. E. (1989). Conservation of resources: a new attempt at conceptualizing stress. Am. Psychol. 44, 513–524. doi: 10.1037/0003-066X.44.3.513, 2648906

[ref11] Hoseiny M. Mahmoodi A. Maredpour A. (2019). Survey relationship of psychological capital with marital intimacy of married students the mediating role of social welfare. Armaghane Danesh Bimonthly J. 24, 388–400.

[ref12] Jang R. Cho S. (2025). Exploring the effects of social comparison and materialism on marriage intention among unmarried young adults: the moderating role of anxiety. Korean J. Soc. Personal Psychol. 39, 99–126. doi: 10.21193/kjspp.2025.39.1.005

[ref13] Li Y. Cheng Q. Teng S. (2025). Exploration of the marriage and love concepts, marriage anxiety, and policy attitudes of unmarried youth in China. Youth Explor. 1, 27–37. doi: 10.13583/j.cnki.issn1004-3780.2025.01.003

[ref14] Li T. Zheng Y. Yan Y. (2022). Has marriage and childbirth in China been institutionalized?—discovery and discussion based on a survey of Chinese college students' views on marriage and childbirth. Women's Stud. Essays 3, 85–102.

[ref15] Liu N. De Winne S. De Cooman R. Smet M. Lattanzi N. (2025). Unraveling the relationship between algorithmic management, leader’s social distance, and employee engagement: an exchange perspective. Int. J. Hum. Resour. Manag. 36, 1373–1406. doi: 10.1080/09585192.2025.2509777

[ref16] Luerssen A. Jhita G. J. Ayduk O. (2017). Putting yourself on the line: self-esteem and expressing affection in romantic relationships. Personal. Soc. Psychol. Bull. 43, 940–956. doi: 10.1177/0146167217702374, 28903707

[ref17] Luthans F. Avolio B. J. Avey J. B. Norman S. M. (2007). Positive psychological capital: measurement and relationship with performance and satisfaction. Pers. Psychol. 60, 541–572. doi: 10.1111/j.1744-6570.2007.00083.x

[ref18] Lv G. Liu M. Yang J. (2024). On the relationship between economic uncertainty and mental health. Mod. Commer. Ind. 45, 161–163. doi: 10.19311/j.cnki.1672-3198.2024.02.053

[ref19] Mao Z. Ji S. Wan L. (2024). The development trends of contemporary college students' views on love, marriage and childbearing under low fertility rate: an analysis based on 32,282 survey data nationwide. Youth Explor. 2, 88–101. doi: 10.13583/j.cnki.issn1004-3780.2024.02.008

[ref20] Miao K. Huang Y. (2022). High expectation mate selection and low fertility trap: sociological reflection on the marriage and childbearing difficulties of contemporary youth. Chin. Youth Res. 5, 44–28. doi: 10.19633/j.cnki.11-2579/d.2022.0064

[ref21] Mullainathan S. Shafir E. (2013). Bookshelf: Scarcity: Why having too little means so much. Sci. News. 184, 34–35. doi: 10.1002/scin.5591840820

[ref22] Nasir A. Javed U. Hagan K. Chang R. Kundi H. Amin Z. . (2025). Social determinants of financial stress and association with psychological distress among young adults 18-26 years in the United States. Front. Public Health 12:1485513. doi: 10.3389/fpubh.2024.1485513, 39845680 PMC11752891

[ref23] Park S. S. Rosén L. A. (2013). The marital scales: measurement of intent, attitudes, and aspects regarding marital relationships. J. Divorce Remarriage 54, 295–312. doi: 10.1080/10502556.2013.780491

[ref24] Shackelford T. K. (2022). “Foundations of evolution,” in The Cambridge Handbook of Evolutionary Perspectives on Sexual Psychology, Charles Crawford (Ed.) (Cambridge: Cambridge University Press), 1–134.

[ref25] Suleman F. Carvalho D. (2024). Young graduates and economic recession: lessons from the pandemic to prevent the (re)incidence of mental health symptoms. Int. J. Health Plann. Manag. 39, 196–203. doi: 10.1002/hpm.3737, 37957781

[ref26] Tian Q. Zhang L. Sun S. (2019). A study on the influence of economic pressure on women's mate preference from the perspective of evolutionary psychology. Psychol. Sci. 42, 681–687. doi: 10.16719/j.cnki.1671-6981.20190325

[ref27] Xu Y. (2020). Research on the marriage and love difficulties of rural children from the perspective of life course. China Youth Stud. 1, 56–119. doi: 10.19633/j.cnki.11-2579/d.2020.0009

[ref28] Yang J. Shi D. (2024). Research on the changes of marriage and love views among Chinese youth in the new era. Youth Explor. 4, 15–29. doi: 10.13583/j.cnki.issn1004-3780.2024.04.002

[ref29] Zhang X. Sun L. Wang Z. Yu G. (2015). The influence of positive psychological capital and love views on subjective feelings of love among college students. Chin. J. Health Psychol. 23, 1812–1816. doi: 10.13342/j.cnki.cjh

